# TREM2 regulates obesity-induced insulin resistance via adipose tissue remodeling in mice of high-fat feeding

**DOI:** 10.1186/s12967-019-2050-9

**Published:** 2019-09-02

**Authors:** Can Liu, Pinhao Li, Hui Li, Sicong Wang, Lifeng Ding, Hanbin Wang, Hui Ye, Yue Jin, Jinchao Hou, Xiangming Fang, Qiang Shu

**Affiliations:** 1 Cardiovascular Surgery, Children’s Hospital, Zhejiang University School of Medicine, No. 3333 Binsheng Road, Hangzhou, 310052 Zhejiang China; 2 The First Affiliated Hospital, Anesthesiology, Zhejiang University School of Medicine, Hangzhou, China

**Keywords:** TREM2, Adipose tissue remodeling, Obesity, Insulin resistance, Diabetes

## Abstract

**Background:**

Adipose tissue remodeling plays a significant role in obesity-induced insulin resistance. Published studies reported that level of trigger receptor expressed on myeloid cells 2 (TREM2) in adipose tissue is up-regulated in animal models of obesity. This study aims to investigate whether TREM2 regulates obesity-induced insulin resistance via modulating adipose tissue remodeling in mice of high-fat diet (HFD).

**Methods:**

Wild-type (WT) and TREM2^−/−^ mice were both fed with a controlled-fat diet (CFD) or HFD for 12 weeks and studied for obesity and insulin resistance. Meanwhile, epididymal adipose tissue (EAT) was examined for morphological and pathological changes to determine adipose tissue remodeling. After that, adipocyte-derived MCP-1 was measured in adipocytes, adipose tissue and circulation. Next, inflammatory cytokines were determined in adipose tissue macrophages (ATM). At last, livers were analyzed for hepatic steatosis.

**Results:**

TREM2^−/−^ mice on HFD had increased obesity and insulin resistance compared with WT counterparts. Adipose tissue from TREM2^−/−^ mice exhibited reduced mass but greater adipocyte hypertrophy and increased adipocyte death. Besides, adipocyte-derived MCP-1 was down-regulated in TREM2^−/−^ mice, and circulating MCP-1 level was lower than that of WT mice. Furthermore, TREM2^−/−^ mice displayed reduced infiltration of F4/80^+^CD11c^+^ macrophages into adipose tissue, which was unable to form crown-like structures (CLS) to clean dead adipocytes and cellular contents. Also, TREM2 deficiency augmented inflammatory response of adipose tissue macrophages in HFD mice. In addition, TREM2^−/−^ mice demonstrated more severe hepatic steatosis than WT counterparts under HFD feeding.

**Conclusions:**

Trigger receptor expressed on myeloid cells 2 may function as a feedback mechanism to curb obesity-induced insulin resistance via regulating adipose tissue remodeling.

## Background

Obesity is highly prevalent all over the world, due to changes in our lifestyle and diet [1, 2]. It is estimated that obesity has affected more than 600 million adults and 100 million children at present [2]. Accumulating evidence has demonstrated that obesity is the most important and common cause of insulin resistance [3, 4]. During obesity, adipose tissue changes the number and size of adipocytes. In the meantime, cells of various types in stromal vascular fraction (SVF) of adipose tissue, especially adipose tissue macrophages (ATM), undergo numerical and functional changes. These biological processes are termed as “adipose tissue remodeling” [5]. Adipose tissue remodeling regulates physiological functions of adipose tissue, which plays a significant role during the pathogenesis and etiology of obesity-induced insulin resistance [5].

Trigger receptor expressed on myeloid cells 2 (TREM2), which belongs to the immunoglobulin superfamily of receptors [6], is mainly expressed on myeloid cells, such as macrophages [7], dendritic cells [8] and microglia [9, 10] for regulating various cell biological behaviors including survival, proliferation, differentiation, phagocytosis and inflammatory response [6–13]. Recent studies have shown that TREM2 is also expressed on mature adipocytes [14]. Furthermore, TREM2 can act as a lipid sensing receptor to recognize and bind lipids [9]. In animal models of obesity, TREM2 gene expression was up-regulated in adipose tissue [14–16]. However, it is unknown whether TREM2 regulates obesity-induced insulin resistance via adipose tissue remodeling. In the present study, we tried to determine the effect of TREM2 gene deficiency on adipose tissue remodeling in mice of high-fat diet (HFD), and explored the effect of TREM2 on obesity-induced insulin resistance. We first examined obesity, insulin resistance and adipose tissue remodeling in TREM2 knockout (TREM2^−/−^) and wild-type (WT) mice under HFD challenge. Then, we determined adipocyte hypertrophy and adipocyte death of epididymal adipose tissue (EAT). Next, we explored numerical changes of macrophages and its underlying mechanism. After that, we measured inflammatory response of adipose tissue macrophages in HFD mice. Finally, we evaluated hepatic steatosis in mice under HFD feeding.

## Materials and methods

### Animals and diets

All animal experiments in this study were approved by the Animal Care and Use Committee of Zhejiang University. WT mice of C57BL/6 were purchased from Shanghai SLAC Laboratory Animal Co. TREM2^−/−^ mice with the background of C57BL/6 were generously provided by Professor Macro Colonna (Department of Pathology and Immunology, School of Medicine) from Washington University in St. Louis [12]. All animals in this research were kept in the Laboratory Animal Center of Zhejiang University under an environmentally controlled condition, with temperature stable at 22 ± 2 °C, humidity stable at 55 ± 5% and a 12/12 h light/dark cycle. Male WT and TREM2^−/−^ mice with the age of 6 weeks and bodyweight of 21.0–23.0 g were both fed with HFD (D12492, 60% kcal of energy from fat, Research Diets) ad libitum for 12 weeks, and control WT and TREM2^−/−^ mice were fed with controlled-fat diet (CFD) (D12450B, 10% kcal of energy from fat, Research Diets). Food consumption was recorded twice a week and bodyweight was monitored weekly.

### Insulin tolerance test (ITT) and glucose tolerance test (GTT)

For ITT, mice were fasted for 6 h before an intraperitoneal injection of insulin with the dosage of 0.8 U/kg bodyweight (for CFD mice) or 1.0 U/kg bodyweight (for HFD mice). For GTT, mice were fasted for 16 h before an intraperitoneal injection of glucose with the dosage of 1.5 g/kg bodyweight. Tail vein blood was collected at 0, 15, 30, 60, 90 and 120 min after injection. Glucose level was measured with a glucometer (Accu-Chek Aviva, Roche Diagnostics).

### Tissue and blood collection

Mice were fasted overnight for 16 h before anesthetizing with 4% chloral hydrate. Blood was collected from retro-orbital venous and spun down at 2500 g for 5 min under 4 °C. After separation, serum was stored under − 80 °C for further analysis. Epididymal fat pads were dissected and weighed. After rinsing in PBS, EAT was divided into three parts: one part was kept in a − 80 °C freezer; one part was fixed in 10% neutral-buffered formalin; while the last part was for adipocyte and macrophage isolation.

### Blood biochemistry analysis

Serum samples stored under − 80 °C were thawed under room temperature. After 5 min of centrifugation at 350*g* under 4 °C, supernatant was collected and kept on ice temporarily. Concentrations of fasting glucose (298-65701, Wako Chemicals), free fatty acid (FFA) (294-63601, Wako Chemicals), triglyceride (F001, Nanjing Jiancheng Bioengineering Institute) and total cholesterol (F002, Nanjing Jiancheng Bioengineering Institute) were measured according to the manufacturer’s instructions. While serum concentrations of MCP-1 were determined using commercially-available ELISA kit (MJE00, R&D).

### Insulin stimulation

To examine insulin signaling pathway activity in adipose tissue, mice were fasted for 6 h before anesthetizing with 4% chloral hydrate. Left EAT (marked as control) was removed, rinsed in PBS and flash frozen in liquid nitrogen. After that, abdominal cavity was closed up temporarily. Insulin was injected intraperitoneally with the dosage of 1.0 U/kg bodyweight for stimulation. Right EAT (marked as insulin-15 min) was harvested 15 min post injection and rinsed in PBS to remove possible contamination of insulin and flash frozen in liquid nitrogen. All EAT samples were transferred to a − 80 °C freezer for Western blotting.

### Adipocyte and macrophage isolation and purification

Adipocyte and macrophage isolation protocol was based on previous publication with a minor modification [17]. Briefly, epididymal fat pads were minced into small pieces before incubating with collagenase type II (17101015, Gibico) on a 37 °C heated shaker for 40 min. Then, cell suspension was passed through a 100 micron filter. After repeated centrifugations of 1000*g*, supernatant layer (floating adipocytes) was collected from top. In the meantime, cell pellet (SVF) was resuspended in ACK Lysing Buffer to remove erythrocytes for further purification of macrophages.

### RT-PCR

Total RNA extraction and RT-PCR were performed as published protocol [18]. The relative gene expression level was normalized to β-actin mRNA expression. Specific primers used in this research for β-actin, TNF-α, IL-1β, IL-6 and iNOS were listed in Table 1.

**Table 1:** Primers sequences for RT-PCR

Gene	Forward (from 5′ to 3′)	Reverse (from 5′ to 3′)
β-actin	CGTTGACATCCGTAAAGACC	AACAGTCCGCCTAGAAGCAC
TNF-α	TACTGAACTTCGGGGTGA	ACTTGGTGGTTTGCTACG
IL-1β	GAAATGCCACCTTTTGACAGTG	TGGATGCTCTCATCAGGACAG
IL-6	CTGCAAGAGACTTCCATCCAG	AGTGGTATAGACAGGTCTGTTGG
iNOS	CAGGCTGGAAGCTGTAACAAAG	GAAGTCATGTTTGCCGTCACTC

### Western blotting

Samples of adipose tissue for detecting activity of insulin signaling pathway and samples of adipocytes for detecting MCP-1 expression were homogenized in cell lysis buffer supplemented with phenylmethylsulfonyl fluoride (PMSF) and protease inhibitor cocktail. After 10 min of incubation on ice, samples were centrifuged at 14,000 rpm under 4 °C for 15 min. Protein layer was extracted by a 1 ml syringe penetrating through floating lipid layer from the top. Protein content was determined by using bicinchoninic acid (BCA) assay. Samples contained 30 μg of protein were separated on 12% Bis–Tris gels (NP0343BOX, Thermo Fisher Scientific). Protein was wet transferred onto polyvinylidene difluoride (PVDF) membranes. Membranes were incubated with specific antibodies against AKT (9272, Cell Signaling Technology), p-AKT (9271, Cell Signaling Technology), MCP-1 (sc-52701, Santa Cruz Biotechnology) and β-tubulin (70-ab009-040, MultiSciences) under 4 °C overnight. All antibodies were diluted 1:1000 in 5% BSA. Membranes were then incubated with corresponding secondary antibodies for 1 h at room temperature. Western blots were developed by enhanced chemiluminescence (20-500-500, Biological Industries) and detected by X-ray films.

### Histology and immunohistochemistry analysis

After fixing in 10% neutral-buffered formalin for 72 h, samples were dehydrated and paraffin embedded. EAT was sectioned into 4 μm sections and stained with hematoxylin and eosin (H&E) for morphological evaluation. The cross sectional area of each adipocyte was quantified by using ImageJ software. To determine live adipocytes, EAT sections were first stained with an antibody to perilipin (20R-PP004, Fitzgerald, 1:200 dilution with PBS) followed by a rabbit anti-guinea pig secondary antibody (ab6771, Abcam, 1:2000 dilution with PBS). Dead adipocytes, defined as adipocytes without positive perilipin expression as published before [19, 20], were counted under random 200× microscopic fields and expressed as the percentage of total adipocytes of each image. To determine macrophage infiltration, EAT sections were first stained with an antibody to F4/80 (MCA497, AbD Serotec, 1:50 dilution with PBS) followed by a goat anti-rat secondary antibody (PV-9004, ZSGB-BIO).

### Flow cytometry (FCM)

After removing erythrocytes, SVF was resuspended in PBS and incubated with FcR Blocking Reagent (130-092-575, Miltenyi Biotec) in the dark on ice for 30 min. Then cells were stained with antibodies of PE-CY7-conjugated anti-F4/80 (25-4801-82, eBioscience), PE-conjugated anti-CD11c (557401, BD) and APC-conjugated anti-CD206 (141708, Biolegend). FCM was performed by using LSRFortessa (BD).

### Statistical analysis

All analyses were calculated with GraphPad Prism6 software. Results were expressed as the mean ± SEM. The statistical significance was identified with one-way ANOVA (Tukey test for post-hoc comparison) and Student’s *t* test. A value of *p* < 0.05 was considered statistically significant.

## Results

### ***TREM2***^***−/−***^*** mice exhibit increased obesity, promoted insulin resistance and altered adipose tissue remodeling under HFD feeding***

**Fig. 1:**
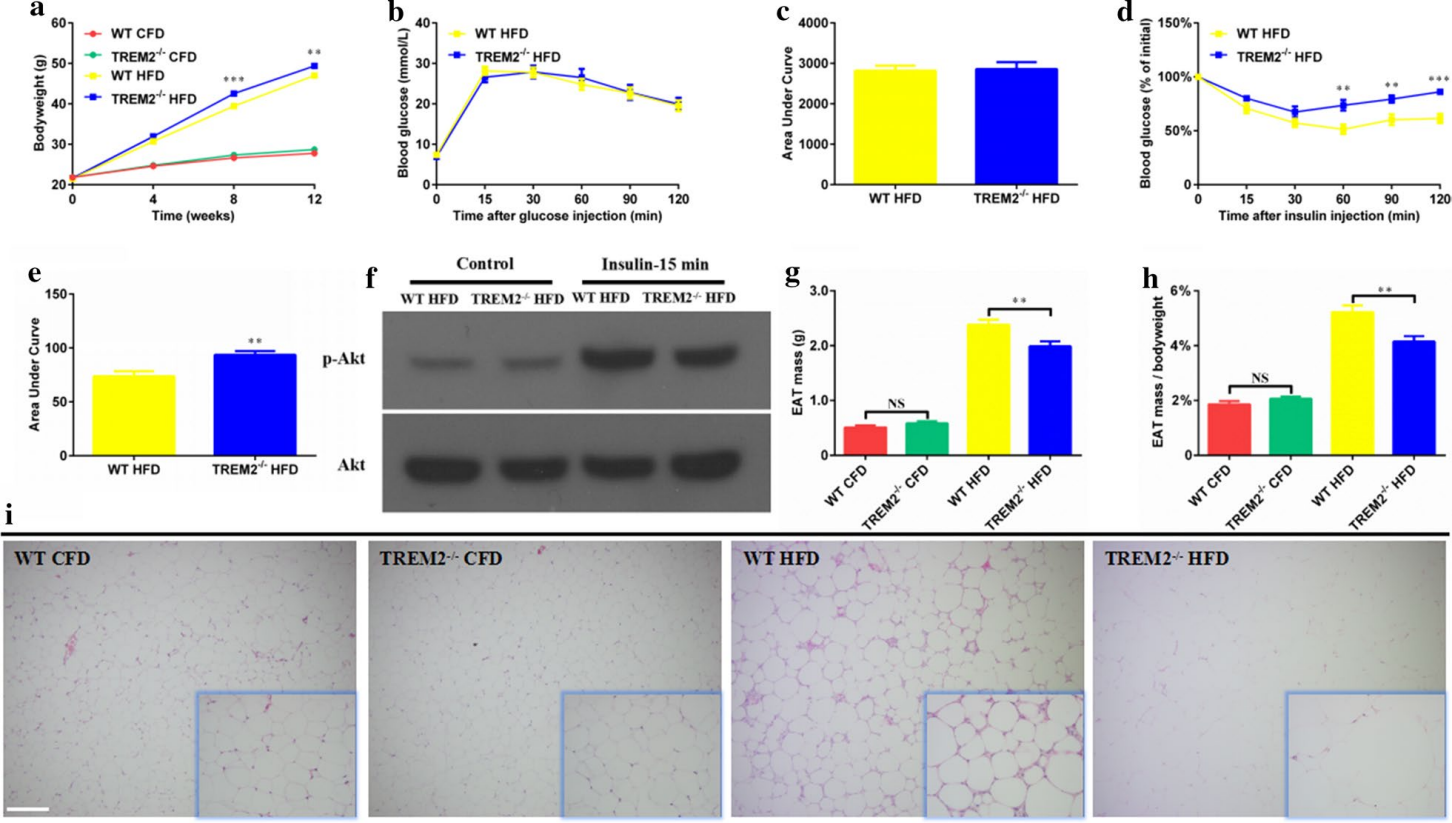
TREM2^−/−^ mice exhibited increased obesity, insulin resistance and altered adipose tissue remodeling under HFD. WT and TREM2^−/−^ mice of C57BL/6 of 6 weeks were fed with CFD or HFD for 12 weeks. **a** Bodyweight of WT and TREM2^−/−^ mice under CFD or HFD feeding (N = 16/group). **b** GTT and **c** AUC of WT and TREM2^−/−^ mice after 12 weeks of HFD feeding (N = 9/group). **d** ITT and **e** AUC of WT and TREM2^−/−^ mice after 12 weeks of HFD feeding (N = 7/group). **f** Protein analysis of Akt and p-Akt in total adipose tissue of HFD feeding mice. **g** Epididymal fat pad mass and **h** proportion of bodyweight of WT and TREM2^−/−^ mice after 12 weeks of CFD (N = 5/group) or HFD (N = 10/group) feeding. **i** Representative images of H&E staining of epididymal adipose tissue of WT and TREM2^−/−^ mice after 12 weeks of CFD or HFD feeding. Original magnification is ×100 and ×400 (within box at bottom right), scale bar = 200 μm. Data are presented as mean ± SEM. *p < 0.05, **p < 0.01, ***p < 0.001, ****p < 0.0001

To clarify the role of TREM2 deficiency on obesity-induced insulin resistance, we fed TREM2^−/−^ mice and their WT counterparts with CFD or HFD for 12 weeks. There was no significant difference in bodyweight between WT and TREM2^−/−^ mice under CFD. However, we observed a higher bodyweight in TREM2^−/−^ mice after HFD feeding compared with WT counterparts (39.4 ± 0.6 g in WT mice vs. 42.6 ± 0.7 g in TREM2^−/−^ mice at 8 weeks, N = 16/group, *p* < 0.001; 47.0 ± 0.4 g in WT mice vs. 49.4 ± 0.5 g in TREM2^−/−^ mice at 12 weeks, N = 16/group, *p* < 0.01) (Fig. 1a). To confirm whether the rise in bodyweight was due to an increased food intake, we compared food consumption of each group. All groups had similar levels of food consumption (*p* > 0.05), indicating the difference in bodyweight was independent of the amount of food intake (Additional file 1: Figure S1). To determine the effect of TREM2 deficiency on insulin resistance, we carried out GTT and ITT in both TREM2^−/−^ and WT mice on CFD and HFD. In CFD mice, TREM2 deficiency didn’t alter glucose levels compared with WT in both GTT and ITT (Additional file 2: Figure S2). When HFD mice were challenged with insulin but not glucose, TREM2^−/−^ mice demonstrated higher blood glucose levels at 60, 90 and 120 min (*p* < 0.01) (Fig. 1b–e) compared with WT mice. Next, we analyzed P-Akt protein levels in EAT of WT and TREM2^−/−^ mice on HFD to determine the activity of insulin signaling pathway. TREM2^−/−^ mice showed lower level of Akt phosphorylation in Ser473 residue, as compared with WT mice, indicating a suppressed insulin signaling (Fig. 1f), which was in accordance with worse ITT results. Consistently, TREM2 deficiency elevated fasting blood glucose levels of HFD mice after 12 weeks of feeding (14.33 ± 0.90 mmol/l in WT mice vs. 17.49 ± 1.19 mmol/l in TREM2^−/−^ mice, N = 13/group, *p* < 0.05) (Additional file 3: Figure S3D). The mass and percentage of bodyweight of epididymal fat pads were comparable between WT and TREM2^−/−^ mice fed with CFD, but were both reduced in HFD-fed TREM2^−/−^ mice compared with WT counterparts (2.38 ± 0.09 g in WT mice vs. 1.99 ± 0.09 g in TREM2^−/−^ mice, N = 10/group, *p* < 0.01) (Fig. 1g) (5.23 ± 0.23% in WT mice vs. 4.15 ± 0.20% in TREM2^−/−^ mice, N = 10/group, *p* < 0.01) (Fig. 1h). The histopathology of EAT showed no difference between WT and TREM2^−/−^ mice on CFD. H&E staining revealed that adipocytes from TREM2^−/−^ mice under HFD feeding were considerably enlarged, while the number of macrophages was significantly reduced with fewer crown-like structures (CLS) (Fig. 1i).

### TREM2^−/−^ mice demonstrated greater adipocyte hypertrophy and adipocyte death under HFD feeding

**Fig. 2:**
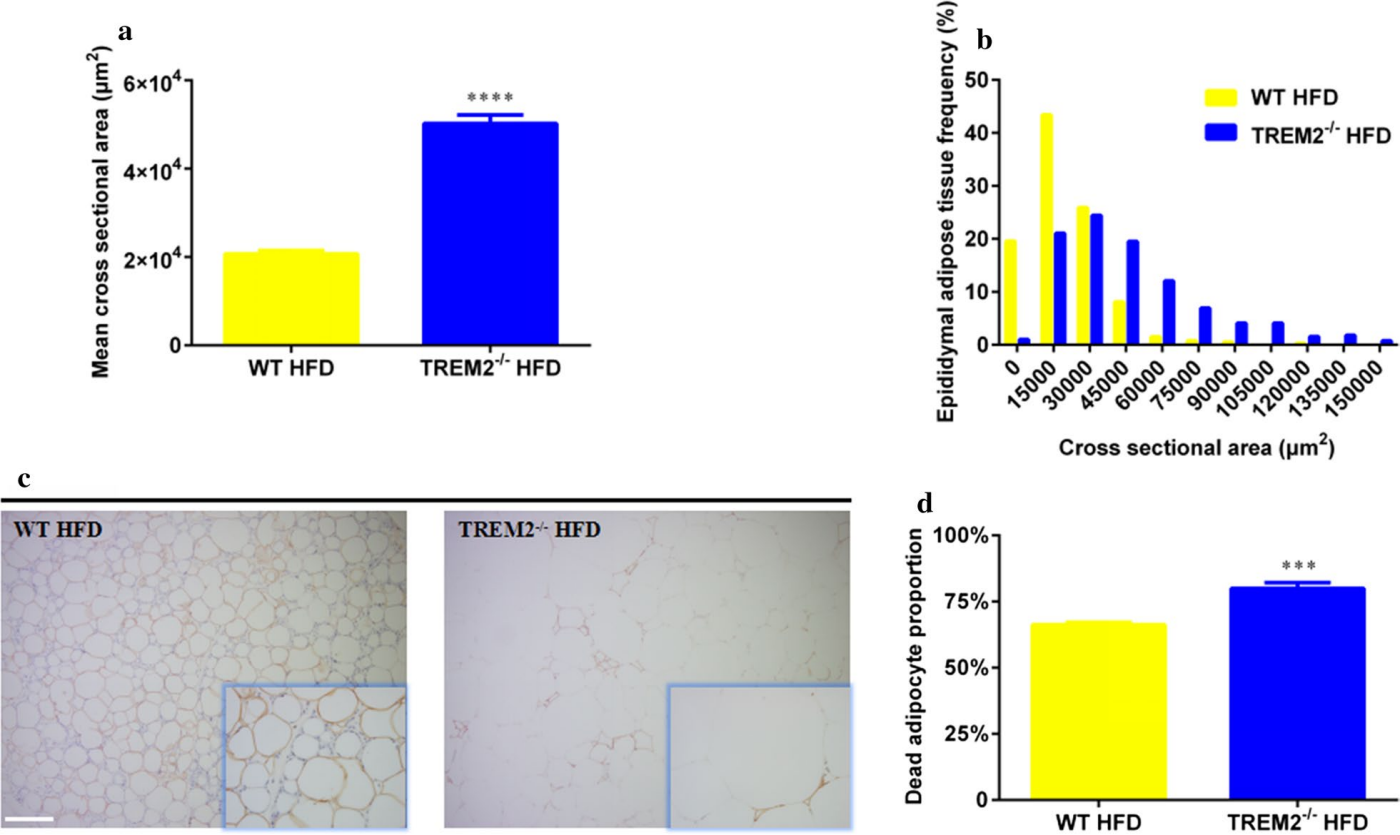
TREM2^−/−^ mice demonstrated greater adipocyte hypertrophy and adipocyte death under HFD. WT and TREM2^−/−^ mice of C57BL/6 of 6 weeks were fed with HFD for 12 weeks. **a** Mean cross sectional area of epididymal adipocytes of WT and TREM2^−/−^ mice after 12 weeks of HFD feeding (N = 394 from WT mice, N = 389 from TREM2^−/−^ mice). **b** Histogram of cross sectional area of epididymal adipocytes of WT and TREM2^−/−^ mice after 12 weeks of HFD feeding. **c** Representative images of perilipin staining of epididymal adipose tissue of WT and TREM2^−/−^ mice after 12 weeks of HFD feeding. Original magnification is ×100 and ×400 (within box at bottom right), scale bar = 200 μm. **d** Dead adipocytes proportion of WT and TREM2^−/−^ mice after 12 weeks of HFD feeding (N = 6/group). Data are presented as mean ± SEM. *p < 0.05, **p < 0.01, ***p < 0.001, ****p < 0.0001

Pathological examination revealed larger adipocytes of EAT from TREM2^−/−^ mice on HFD. By calculating the cross sectional areas, adipocytes from TREM2^−/−^ mice were significantly enlarged (2.07 ± 0.08 × 10^4^ μm^2^ of WT mice vs. 5.02 ± 0.20 × 10^4^ μm^2^ of TREM2^−/−^ mice, N = 394 from WT mice, N = 389 from TREM2^−/−^ mice, *p* < 0.0001) (Fig. 2a). Frequency distribution revealed a higher frequency of larger adipocytes in EAT of TREM2^−/−^ mice (25% percentile: 0.92 × 10^4^ μm^2^ of WT mice vs. 2.50 × 10^4^ μm^2^ of TREM2^−/−^ mice; median: 1.73 × 10^4^ μm^2^ of WT mice vs. 3.98 × 10^4^ μm^2^ of TREM2^−/−^ mice; 75% percentile: 2.99 × 10^4^ μm^2^ of WT mice vs. 6.32 × 10^4^ μm^2^ of TREM2^−/−^ mice, N = 394 from WT mice, N = 389 from TREM2^−/−^ mice) (Fig. 2b), indicating TREM2^−/−^ mice have developed greater adipocyte hypertrophy. Adipocyte hypertrophy has been reported to drive adipocyte death, resulting in adipose tissue dysfunction and inflammation in the end [21]. To determine whether TREM2 deficiency will promote adipocyte death in HFD mice, EAT was immunohistochemically stained for perilipin, an adipocyte-specific cytomembrane protein. Immunohistochemistry revealed that there were fewer perilipin positive adipocytes in EAT of TREM2^−/−^ mice compared with WT counterparts, indicating a promoted adipocyte death (66.22 ± 0.90% of WT mice vs. 79.97 ± 2.16% of TREM2^−/−^ mice, N = 6/group, *p* < 0.001) (Fig. 2c, d). In addition, we observed less macrophage infiltration surrounding dead adipocytes in TREM2^−/−^ mice, so as CLS formation. Taken together, these results demonstrated that TREM2 deficiency promoted epididymal adipocyte hypertrophy and adipocyte death without stimulating macrophage infiltration in HFD mice.

### Adipose tissue macrophages were reduced and unable to form CLS in TREM2^−/−^ mice under HFD feeding

**Fig. 3:**
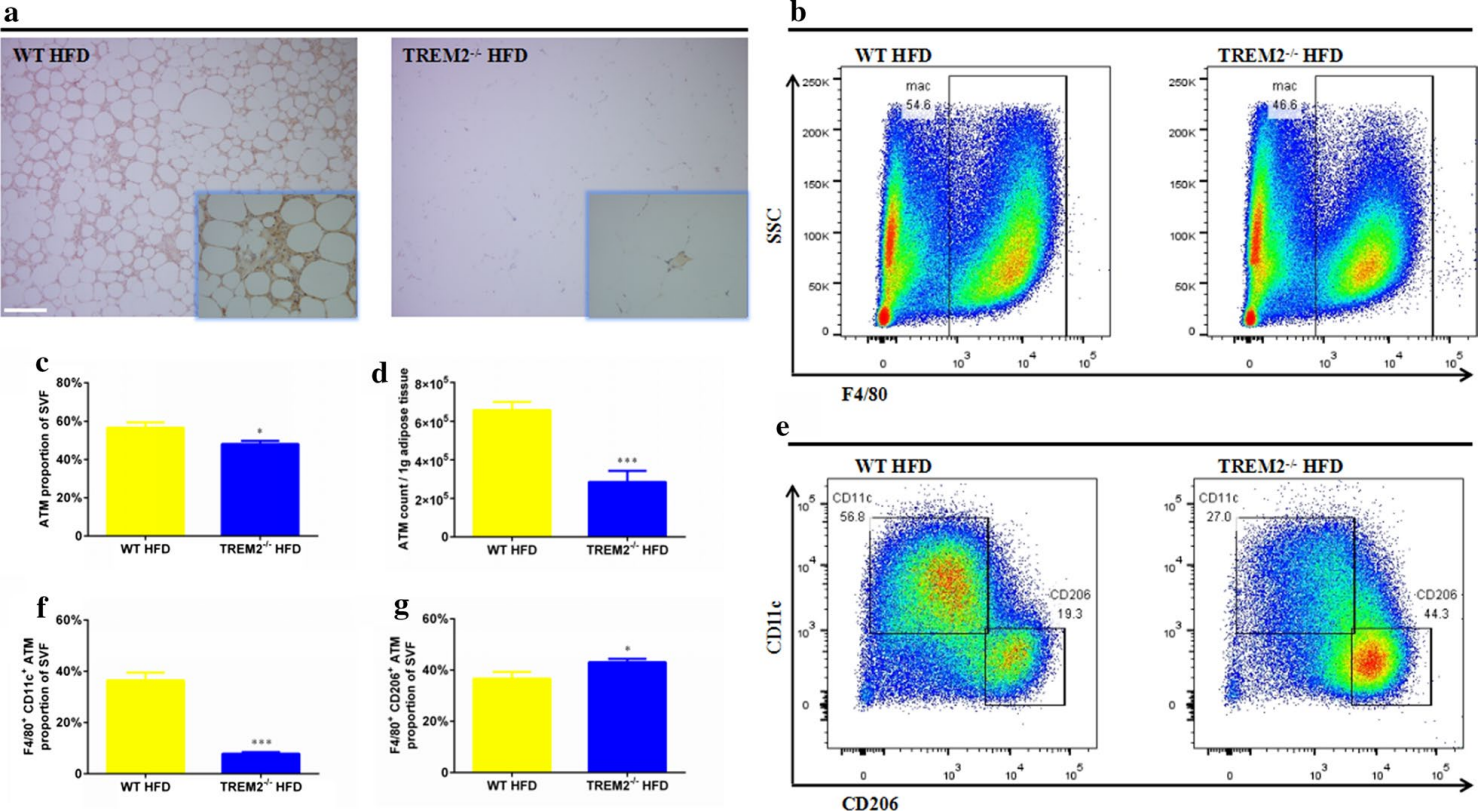
Adipose tissue macrophages were reduced and unable to form CLS in TREM2^−/−^ mice under HFD. WT and TREM2^−/−^ mice of C57BL/6 of 6 weeks were fed with CFD or HFD for 12 weeks. **a** Representative images of F4/80 staining of epididymal adipose tissue of WT and TREM2^−/−^ mice after 12 weeks of HFD feeding. Original magnification is ×100 and ×400 (within box at bottom right), scale bar = 200 μm. **b** Representative images of FCM analysis of macrophages of WT and TREM2^−/−^ mice after 12 weeks of HFD feeding. **c** Macrophage proportion and **d** macrophage count of epididymal adipose tissue of WT and TREM2^−/−^ mice after 12 weeks of HFD feeding (N = 8/group). **e** Representative images of FCM analysis of macrophage phenotypes of WT and TREM2^−/−^ mice after 12 weeks of HFD feeding. Proportion of F4/80^+^CD11c^+^ (**f**) and F4/80^+^CD206^+^ (**g**) macrophages of WT and TREM2^−/−^ mice after 12 weeks of HFD feeding (N = 9/group). Data are presented as mean ± SEM. *p < 0.05, **p < 0.01, ***p < 0.001, ****p < 0.0001

Macrophages play a major role in maintaining normal morphology and function of adipose tissue. In lean subjects, adipose tissue macrophages are mainly F4/80^+^CD206^+^ macrophages scattered within adipose tissue. As obesity develops, F4/80^+^CD11c^+^ macrophages increase encircling dead adipocytes and forming CLS to clean dead adipocytes and cellular contents [22]. We observed less macrophage infiltration and CLS formation in TREM2^−/−^ mice (Fig. 1i). In addition, macrophages surrounding dead adipocytes were considerably reduced in epididymal adipose tissue of TREM2 deficiency mice (Fig. 2c). To verify the effect of TREM2 deficiency on macrophage infiltration and CLS formation, F4/80^+^ staining was used. Consistent with morphological results mentioned above, F4/80^+^ staining revealed reduced macrophage infiltration and CLS formation in TREM2^−/−^ mice under HFD feeding (Fig. 3a). From FCM analysis, we observed the proportion of macrophages in SVF was reduced in TREM2^−/−^ mice (56.69 ± 2.80% of WT mice vs. 48.14 ± 1.67% of TREM2^−/−^ mice, N = 8/group, *p* < 0.05) (Fig. 3c), with reduced total macrophage count (6.57 ± 0.42 × 10^5^/g adipose tissue from WT mice vs. 2.85 ± 0.58 × 10^5^/g adipose tissue from TREM2^−/−^ mice, *p* < 0.001) (Fig. 3d). Having confirmed the effect of TREM2 deficiency on macrophage infiltration in response to a HFD challenge, we wanted to know which phenotype of macrophage was reduced in adipose. FCM analysis revealed a reduced proportion of F4/80^+^CD11c^+^ macrophages (36.45 ± 3.10% of WT mice vs. 7.80 ± 0.62% of TREM2^−/−^ mice, N = 9/group, *p* < 0.001) (Fig. 3f) and an increased proportion of F4/80^+^CD206^+^ macrophages in SVF (36.69 ± 2.61% of WT mice vs. 43.08 ± 1.30% of TREM2^−/−^ mice, N = 9/group, *p* < 0.05) (Fig. 3g).

### TREM2 deficiency down-regulated MCP-1 expression of adipocytes in mice of HFD feeding

**Fig. 4:**
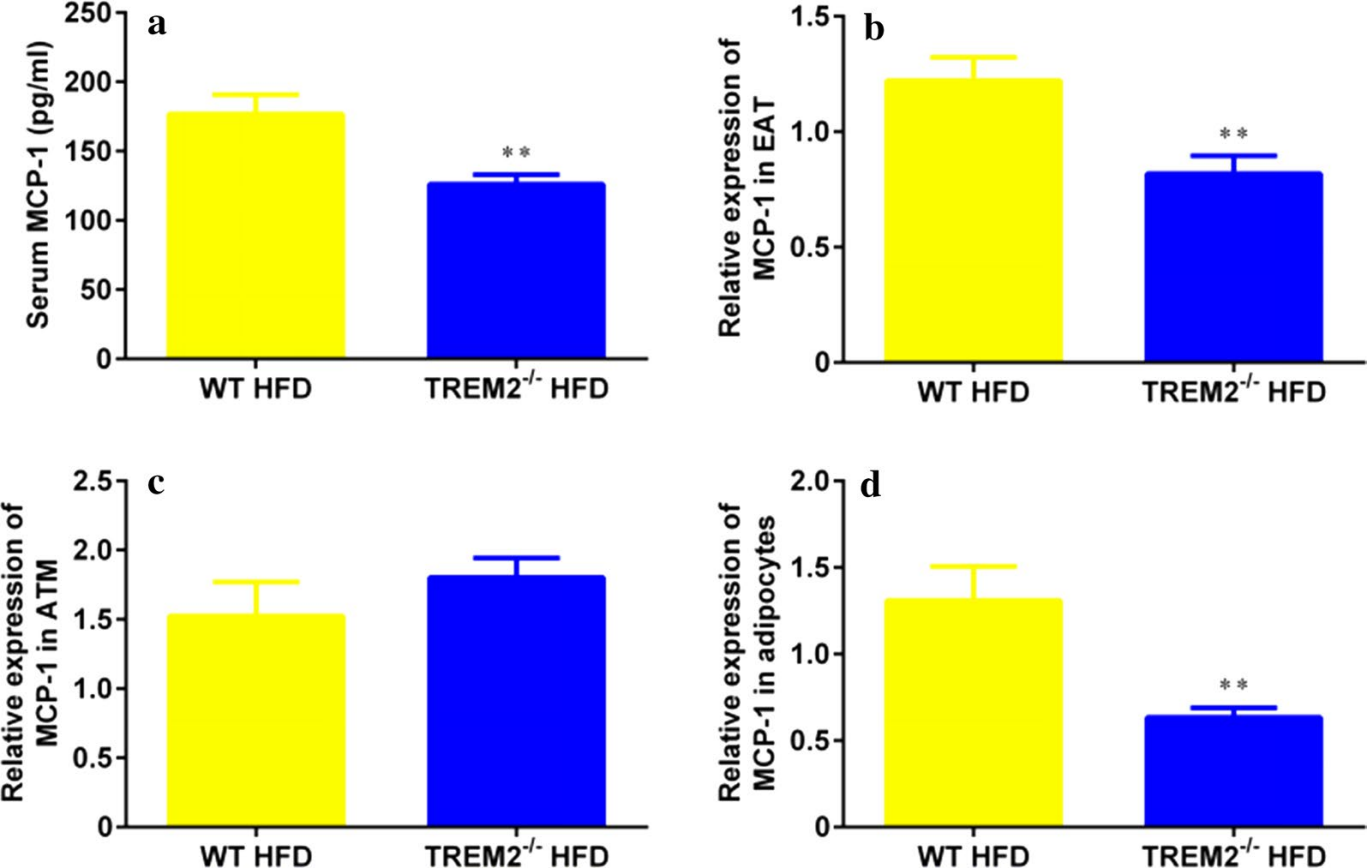
TREM2 deficiency down-regulated MCP-1 expression of adipocytes in mice of HFD. WT and TREM2^−/−^ mice of C57BL/6 of 6 weeks were fed with HFD for 12 weeks. **a** Circulating levels of MCP-1 of WT and TREM2^−/−^ mice after 12 weeks of HFD feeding (N = 19/group). **b** MCP-1 expression levels of adipose tissue of WT and TREM2^−/−^ mice after 12 weeks of HFD feeding (N = 8/group). **c** MCP-1 expression levels of macrophages of WT and TREM2^−/−^ mice after 12 weeks of HFD feeding (N = 8/group). **d** MCP-1 expression levels of adipocytes of WT and TREM2^−/−^ mice after 12 weeks of HFD feeding (N = 8/group). Data are presented as mean ± SEM. *p < 0.05, **p < 0.01, ***p < 0.001, ****p < 0.0001

In obese state, monocytes were recruited from circulation and differentiated into F4/80^+^CD11c^+^ macrophages. Among all kinds of circulating chemokines released by adipose tissue, MCP-1 plays a significant role [23]. To explore whether suppressed F4/80^+^CD11c^+^ macrophage infiltration was due to lower concentration of MCP-1 in blood stream, circulating MCP-1 levels were detected via ELISA. Compared with WT mice, circulating MCP-1 level was significantly lower in TREM2^−/−^ mice on HFD (176.8 ± 13.9 pg/ml of WT mice vs. 126.1 ± 7.0 pg/ml of TREM2^−/−^ mice, N = 19/group, *p* < 0.01) (Fig. 4a). After that, MCP-1 expression was measured with RT-PCR in EAT of WT and TREM2^−/−^ mice. From RT-PCR analysis, we observed a reduced MCP-1 expression in TREM2^−/−^ mice (Fig. 4b). In adipose tissue, both macrophages and adipocytes can generate and secrete MCP-1. Therefore, MCP-1 expression was examined in both macrophages and adipocytes. It turned out that macrophages derived MCP-1 was comparable in both groups (Fig. 4c), while adipocytes MCP-1 expression was down-regulated in TREM2^−/−^ mice at both mRNA level (Fig. 4d) and protein level (Additional file 4: Figure S4). Taken together, these results demonstrated that TREM2 deficiency down-regulated adipocyte-derived MCP-1 expression, leading to lower concentration of circulating MCP-1, which has a reduced effect to recruit monocytes infiltration.

### TREM2 deficiency enhanced macrophage inflammatory response in mice of HFD feeding

**Fig. 5:**
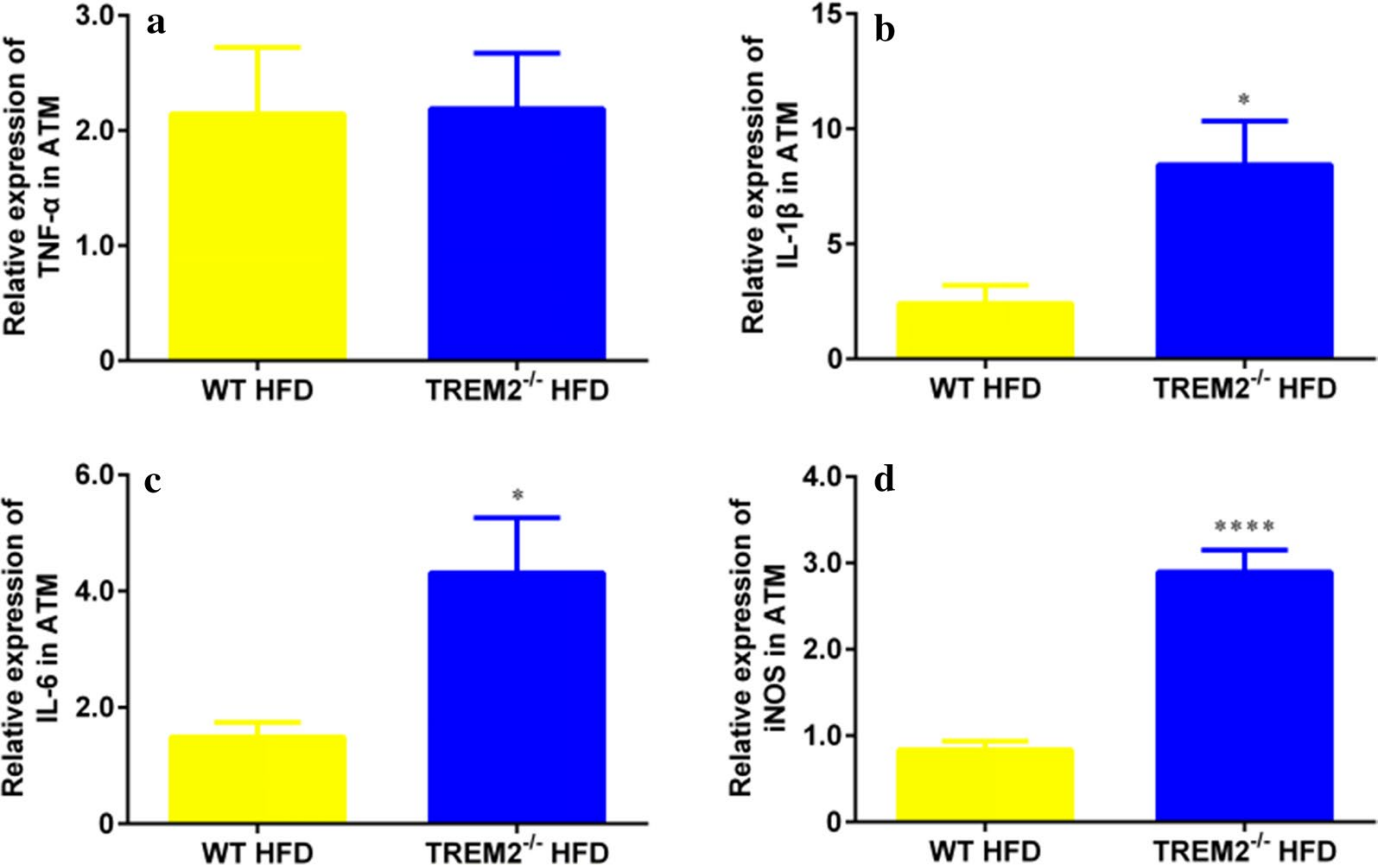
TREM2 deficiency up-regulated macrophage pro-inflammatory cytokine expressions of macrophages in mice of HFD. WT and TREM2^−/−^ mice of C57BL/6 of 6 weeks were fed with HFD for 12 weeks. Inflammatory cytokines were measured with RT-PCR. Relative expression levels of **a** TNF-α, **b** IL-1β, **c** IL-6 and **d** iNOS of macrophages of epididymal adipose tissue of WT and TREM2^−/−^ mice after 12 weeks of HFD feeding (N = 8/group). Data are presented as means ± SEM. *p < 0.05, **p < 0.01, ***p < 0.001, ****p < 0.0001

Macrophages are the main source of pro-inflammatory cytokines in adipose tissue and play a pivotal role in the development of obesity-induced insulin resistance [24]. TREM2 has been known to exert anti-inflammatory effects via attenuating macrophage activation [12, 13]. We proposed that TREM2 deficiency may up-regulate adipose tissue macrophage inflammatory response in mice of HFD feeding. To test this hypothesis, we isolated macrophages from epididymal fat pads of obese WT and TREM2^−/−^ mice to measure pro-inflammatory cytokines. Compared with WT counterparts, adipose tissue macrophages from TREM2^−/−^ mice underwent HFD expressed similar level of TNF-α (Fig. 5a), but higher levels of IL-1β (Fig. 5b), IL-6 (Fig. 5c), and iNOS (Fig. 5d), indicating an enhanced inflammatory response.

### TREM2^−/−^ mice demonstrated more severe hepatic steatosis under HFD feeding

**Fig. 6:**
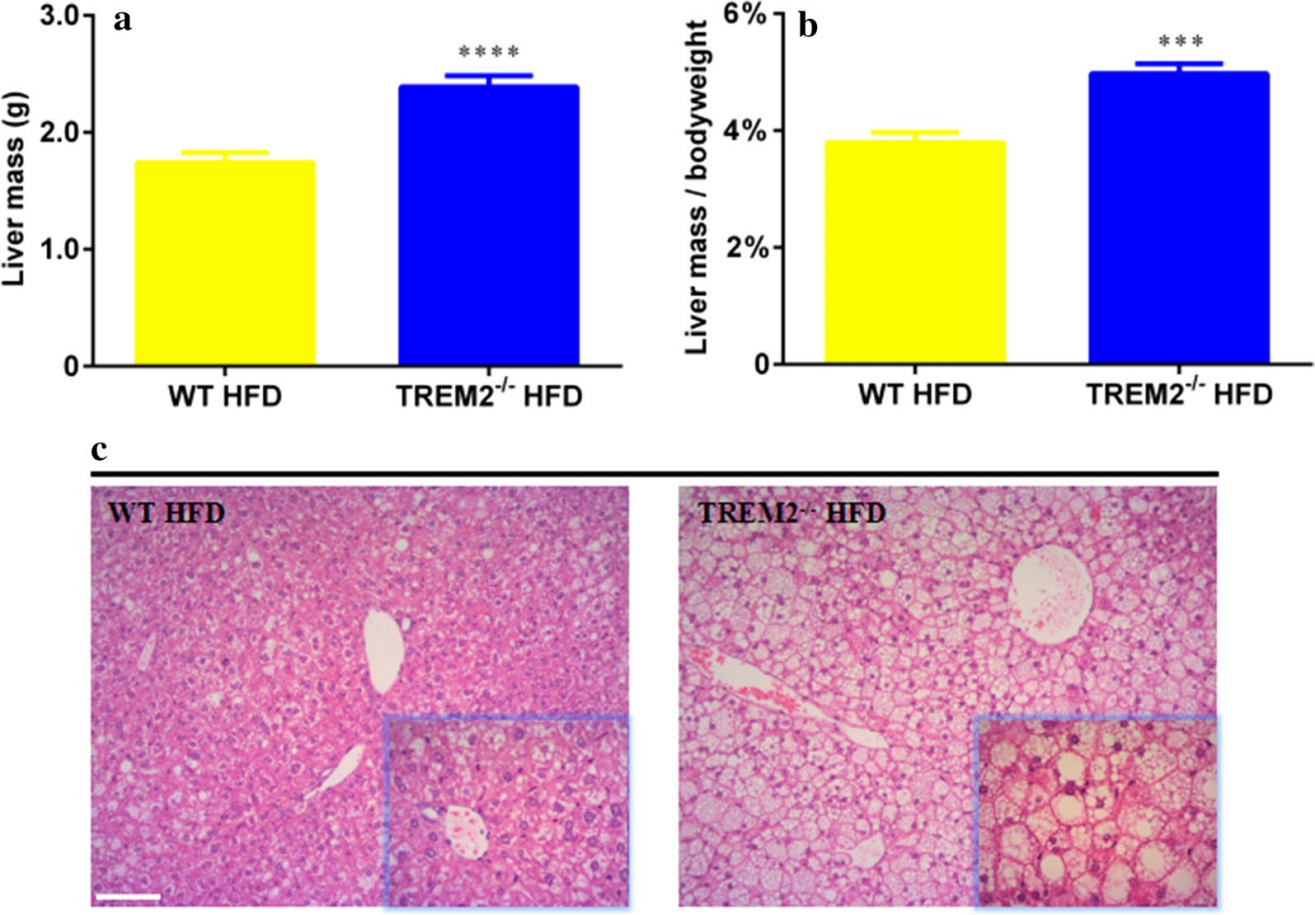
TREM2^−/−^ mice demonstrated more severe hepatic steatosis under HFD feeding. WT and TREM2^−/−^ mice of C57BL/6 of 6 weeks were fed with HFD for 12 weeks. **a** Liver mass and **b** proportion of bodyweight of WT and TREM2^−/−^ mice after 12 weeks of HFD (N = 10/group) feeding. **c** Representative images of H&E staining of liver of WT and TREM2^−/−^ mice after 12 weeks of HFD feeding. Original magnification is ×100 and ×400 (within box at bottom right), scale bar = 200 μm. Data are presented as mean ± SEM. *p < 0.05, **p < 0.01, ***p < 0.001, ****p < 0.0001

It has been reported, loss of EAT is associated with hepatomegaly due to steatosis in mice of chronic obesity [20]. In present study, we observed an increased bodyweight (Fig. 1a) and decreased EAT mass (Fig. 1g, h) in TREM2^−/−^ mice fed with HFD. We speculated that livers from TREM2^−/−^ mice on HFD might demonstrate more severe steatosis compared with WT counterparts. The mass and percentage of bodyweight of livers were both increased in HFD-fed TREM2^−/−^ mice (1.74 ± 0.09 g in WT mice vs. 2.39 ± 0.10 g in TREM2^−/−^ mice, N = 10/group, *p* < 0.0001) (Fig. 6a) (3.79 ± 0.18% in WT mice vs. 4.97 ± 0.18% in TREM2^−/−^ mice, N = 10/group, *p* < 0.001) (Fig. 6b). The histopathology of livers showed more severe hepatic steatosis in TREM2^−/−^ mice than WT mice on HFD (Fig. 6c), while in CFD mice TREM2 deficiency failed to promote hepatic steatosis (Additional file 5: Figure S5).

## Discussion

Our experiments demonstrated that TREM2^−/−^ mice under HFD manifested with increased obesity, insulin resistance and altered adipose tissue remodeling as compared with the WT counterparts. TREM2 deficiency promoted adipocyte hypertrophy and adipocyte death in EAT in HFD mice. Besides, down-regulation of adipocytes-derived MCP-1 expression from TREM2^−/−^ mice lead to suppressed F4/80^+^CD11c^+^ macrophage infiltration and CLS formation. Furthermore, inflammatory response was elevated in macrophages in TREM2^−/−^ mice. In addition, TREM2^−/−^ mice on HFD exhibited more severe hepatic steatosis. Published articles have revealed that in animal models of obesity, TREM2 gene expression was up-regulated in adipose tissue [14–16]. Thus, we hypothesize that TREM2 may act as a feedback mechanism to curb HFD-induced adipose tissue remodeling. These results suggest that TREM2 plays a critical role during the pathogenesis of obesity-induced insulin resistance via regulating adipose tissue remodeling.

Recently, Park [14] has reported that mature adipocytes express TREM2 to regulate adipogenesis. In his study, blocking TREM2 with TREM2-Ig suppressed DMI-induced 3T3-L1 preadipocytes and primary mouse embryonic fibroblasts differentiation into mature adipocytes. When adipogenesis was suppressed during obesity, adipocytes underwent adipocyte hypertrophy (to increase the size of adipocytes instead of number) to meet the demand of energy intake [25]. In our study, we observed enlarged adipocytes in EAT of TREM2^−/−^ mice on HFD (Fig. 1i), with a mean cross sectional area reaching 5.02 ± 0.20 × 10^4^ μm^2^ as compared with 2.07 ± 0.08 × 10^4^ μm^2^ of WT mice (Fig. 2a). Besides, frequency distribution revealed a higher frequency of larger adipocytes in EAT of TREM2^−/−^ mice (25% percentile: 0.92 × 10^4^ μm^2^ of WT mice vs. 2.50 × 10^4^ μm^2^ of TREM2^−/−^ mice; median: 1.73 × 10^4^ μm^2^ of WT mice vs. 3.98 × 10^4^ μm^2^ of TREM2^−/−^ mice; 75% percentile: 2.99 × 10^4^ μm^2^ of WT mice vs. 6.32 × 10^4^ μm^2^ of TREM2^−/−^ mice) (Fig. 2b), indicating TREM2^−/−^ mice have developed greater adipocyte hypertrophy, which can be explained by suppressed adipogenesis [25].

Adipocyte hypertrophy is an important stress factor leading to adipocyte death; besides, hypertrophic adipocytes show features of necrosis as membrane rupture and functional membrane protein loss [19, 20]. In our study, TREM2^−/−^ mice displayed higher incidence of adipocyte death (79.97 ± 2.16% of TREM2^−/−^ mice vs. 66.22 ± 0.90% of WT mice) (Fig. 2c, d), which could be a consequence of greater adipocyte hypertrophy.

Impaired adipogenesis or adipocyte differentiation can bring about a rare medical condition termed as lipodystrophy. Lipodystrophy is characterized by complete or partial loss of adipose tissue, hepatic steatosis and insulin resistance [26]. Similar phenomena were observed in our study. Mass of EAT (1.99 ± 0.09 g in TREM2^−/−^ mice vs. 2.38 ± 0.09 g in WT mice) (Fig. 1g) and its proportion to bodyweight (4.15 ± 0.20% in TREM2^−/−^ mice vs. 5.23 ± 0.23% in WT mice) (Fig. 1h) were both reduced in TREM2^−/−^ mice. Meanwhile, Mass of livers (1.74 ± 0.09 g in WT mice vs. 2.39 ± 0.10 g in TREM2^−/−^ mice) (Fig. 6a) and their proportion to bodyweight (3.79 ± 0.18% in WT mice vs. 4.97 ± 0.18% in TREM2^−/−^ mice) (Fig. 6b) were both increased in TREM2^−/−^ mice. In addition, histopathology confirmed more severe hepatic steatosis in TREM2^−/−^ mice (Fig. 6c). Besides, TREM2^−/−^ mice under HFD feeding demonstrated more severe insulin resistance (Fig. 1d–f and Additional file 3: Figure S3D). We speculate that in mice of HFD feeding, adipogenesis is suppressed due to loss of TREM2, which leads to lipodystrophy.

Under obese state, adipose tissue generates a series of chemokines, among them MCP-1 plays a major role [23]. MCP-1 is released into bloodstream to recruit monocyte infiltration from circulation. In adipose tissue, monocytes were induced into F4/80^+^CD11c^+^ macrophages, which surround dead adipocytes and form CLS to isolate and clear dead adipocytes and cellular contents [22]. In this study, we observed down-regulation of MCP-1 expression in adipocytes from TREM2^−/−^ mice (Fig. 4d and Additional file 4: Figure S4). Lower circulating MCP-1 levels in TREM2^−/−^ mice (126.1 ± 7.0 pg/ml of TREM2^−/−^ mice vs. 176.8 ± 13.9 pg/ml of WT mice) (Fig. 4a) were not sufficient to drive monocyte migration. Therefore, F4/80^+^CD11c^+^ macrophages were reduced in TREM2^−/−^ mice as compared to their WT counterparts (7.80 ± 0.62% of TREM2^−/−^ mice vs. 36.45 ± 3.10% of WT mice) (Fig. 3e, f). Besides, CLS formation was suppressed in EAT of TREM2^−/−^ mice (Figs. 1i, 2c and 3a).

Macrophages are the main source of pro-inflammatory cytokines in adipose tissue and play a pivotal role in the development of obesity-induced insulin resistance [24]. TREM2 has been known as an anti-inflammatory regulator in immune process, since it can suppress inflammatory response via blocking Toll-like receptor signaling pathway [12, 13]. In our study, we observed that macrophages of EAT expressed more pro-inflammatory cytokines such as IL-1β, IL-6 and iNOS in TREM2 knockout mice (Fig. 5).

Published work demonstrated that, down-regulation of TREM2 in adipose tissue in morbid obese patients is associated with advanced insulin resistance [27], which was in consistent with our experiment (Fig. 1d–f and Additional file 3: Figure S3D). Besides, elevated TREM2 expression was observed in obese animal models [14–16]. Hence, we hypothesize that TREM2 may act as a feedback protective mechanism to curb obesity induced-insulin resistance via regulating adipose tissue remodeling. First, TREM2 alleviates adipocyte hypertrophy and adipocyte death via promoting adipogenesis. Next, TREM2 up-regulates adipocyte-derived MCP-1 expression to recruit F4/80^+^CD11c^+^ macrophage infiltration to isolate and clear dead adipocytes and cellular contents. In addition, TREM2 attenuates inflammatory response of macrophages in EAT under HFD feeding.

The present study has one major limitation that should be addressed. Our TREM2^−/−^ mice with the background of C57BL/6 were created according to traditional gene knockout technology [12]. In short, a portion of the trans-membrane and cytoplasmic domains encoded by exons 3 and 4 was deleted in embryonic stem cells [12]. Because all cells in TREM2^−/−^ mice were TREM2 deficient, we can not distinguish whether TREM2 expressed on ATM or adipocytes plays a more important role in the pathogenesis and etiology of obesity-induced insulin resistance. Besides, traditional gene knockout technology allows for the possibilities that TREM2 expression on other tissue cells (yet to be discovered) may influence experimental results. Thus, an animal model with TREM2 conditional knockout (cell-specific knockout) in adipocytes and/or macrophages is warranted in future experiments to delineate the effect of TREM2 on obesity induced insulin resistance.

## Conclusion

In conclusion, our data demonstrated that TREM2 may function as a feedback mechanism to inhibit obesity-induced insulin resistance. Our study suggested that TREM2 may act as a novel biomarker and potential therapeutic target of obesity and insulin resistance.

## Supplementary information

**Additional file 1: Figure S1.** WT and TREM2^−/−^ mice consumed the same amount of food. WT and TREM2^−/−^ mice of C57BL/6 of 6 weeks were fed with CFD or HFD for 12 weeks (N = 10/group) and food intake was monitored weekly. Data are presented as means ± SEM. *p < 0.05, **p < 0.01, ***p < 0.001, ****p < 0.0001.

**Additional file 2: Figure S2.** TREM2 deficiency didn’t alter GTT and ITT in mice of CFD. WT and TREM2^−/−^ mice of C57BL/6 of 6 weeks were fed with CFD for 12 weeks. (A) GTT and (B) AUC of WT and TREM2^−/−^ mice after 12 weeks of CFD feeding (N = 9/group). (C) ITT and (D) AUC of WT and TREM2^−/−^ mice after 12 weeks of CFD feeding (N = 9/group). Data are presented as means ± SEM. *p < 0.05, **p < 0.01, ***p < 0.001, ****p < 0.0001.

**Additional file 3: Figure S3.** TREM2^−/−^ mice demonstrated elevated fasting serum glucose under HFD. WT and TREM2^−/−^ mice of C57BL/6 of 6 weeks (n = 13/group) were fed with HFD for 12 weeks. After HFD challenge, mice were sacrificed after 16 hours of fasting. Serum was collected and metabolic profiles were analyzed. Data are presented as mean ± SEM. *p < 0.05, **p < 0.01, ***p < 0.001, ****p < 0.0001.

**Additional file 4: Figure S4.** Adipocytes from TREM2^−/−^ mice exhibited with down-regulated of MCP-1 level under HFD. WT and TREM2^−/−^ mice of C57BL/6 of 6 weeks were fed with HFD for 12 weeks. After HFD challenge, mice were sacrificed and adipocytes were isolated for detecting MCP-1 protein level.

**Additional file 5: Figure S5.** TREM2 deficiency fail to promote hepatic steatosis in mice of CFD. WT and TREM2^−/−^ mice of C57BL/6 of 6 weeks were fed with CFD for 12 weeks. After CFD challenge, mice were sacrificed and hepatic steatosis was examined via H&E staining. Original magnification is 100× and 400× (within box at bottom right), scale bar = 200 μm.

## Data Availability

The datasets used and/or analysed during the current study are available from the corresponding author on reasonable request.

## Abbreviations

TREM2: trigger receptor expressed on myeloid cells 2
HFD: high-fat diet
CFD: controlled-fat diet
WT: wild-type
EAT: epididymal adipose tissue
CLS: crown-like structures
SVF: stromal vascular fraction
ITT: insulin tolerance test
GTT: glucose tolerance test
FFA: free fatty acid
FCM: flow cytometry
H&E: hematoxylin and eosin
ATM: adipose tissue macrophages
PMSF: phenylmethylsulfonyl fluoride
BCA: bicinchoninic acid
