# Supplementary figures and images for: TREM2 regulates obesity-induced insulin resistance via adipose tissue remodeling in mice of high-fat feeding
Source: J Transl Med. 2019 Sep 2;17:300. doi: 10.1186/s12967-019-2050-9 (PMC6720981; doi:10.1186/s12967-019-2050-9)

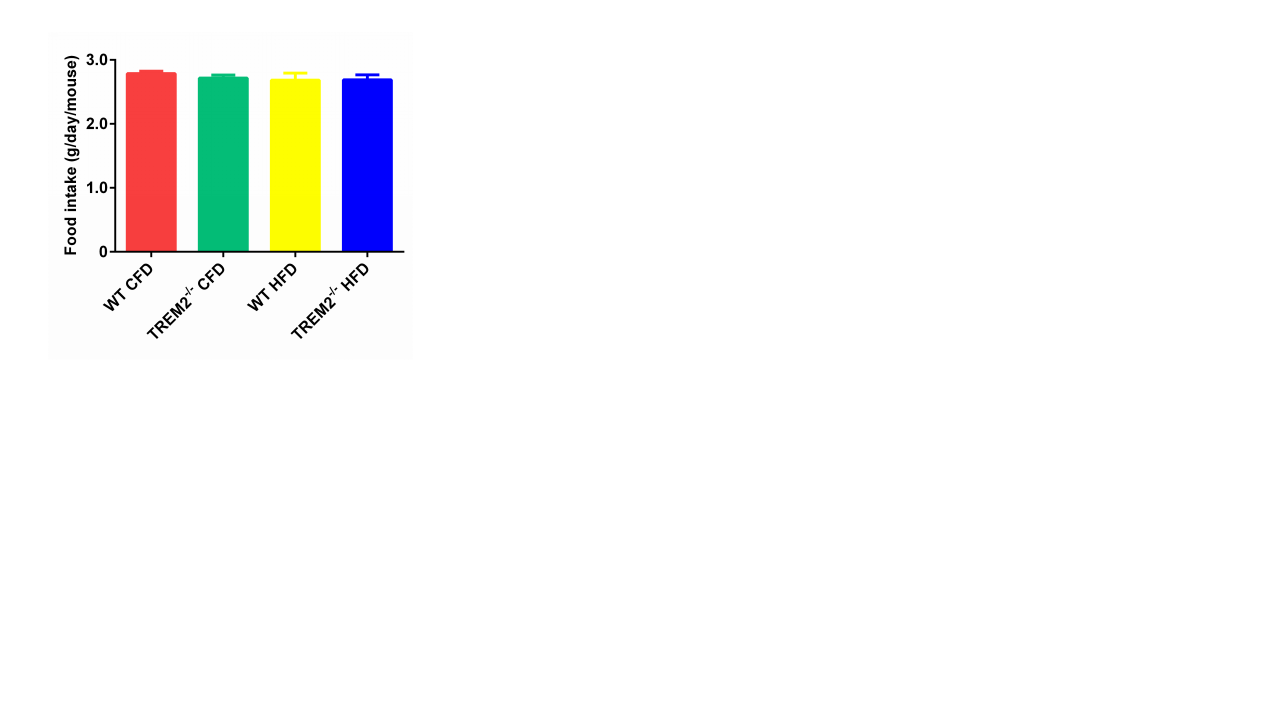

Supplement: Supplementary file 1 — Additional file 1: Figure S1. WT and TREM2−/− mice consumed the same amount of food. WT and TREM2−/− mice of C57BL/6 of 6 weeks were fed with CFD or HFD for 12 weeks (N = 10/group) and food intake was monitored weekly. Data are presented as means ± SEM. *p < 0.05, **p < 0.01, ***p < 0.001, ****p < 0.0001. [file 12967_2019_2050_MOESM1_ESM.tif]

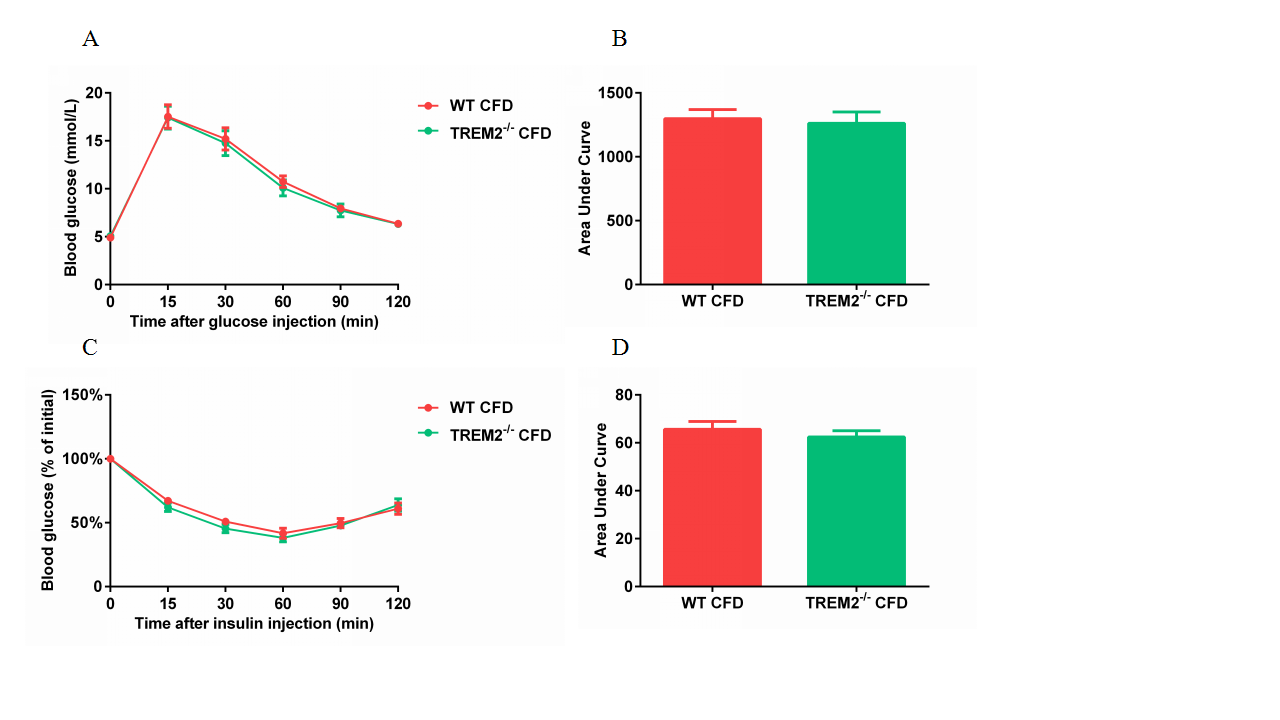

Supplement: Supplementary file 2 — Additional file 2: Figure S2. TREM2 deficiency didn’t alter GTT and ITT in mice of CFD. WT and TREM2−/− mice of C57BL/6 of 6 weeks were fed with CFD for 12 weeks. (A) GTT and (B) AUC of WT and TREM2−/− mice after 12 weeks of CFD feeding (N = 9/group). (C) ITT and (D) AUC of WT and TREM2−/− mice after 12 weeks of CFD feeding (N = 9/group). Data are presented as means ± SEM. *p < 0.05, **p < 0.01, ***p < 0.001, ****p < 0.0001. [file 12967_2019_2050_MOESM2_ESM.tif]

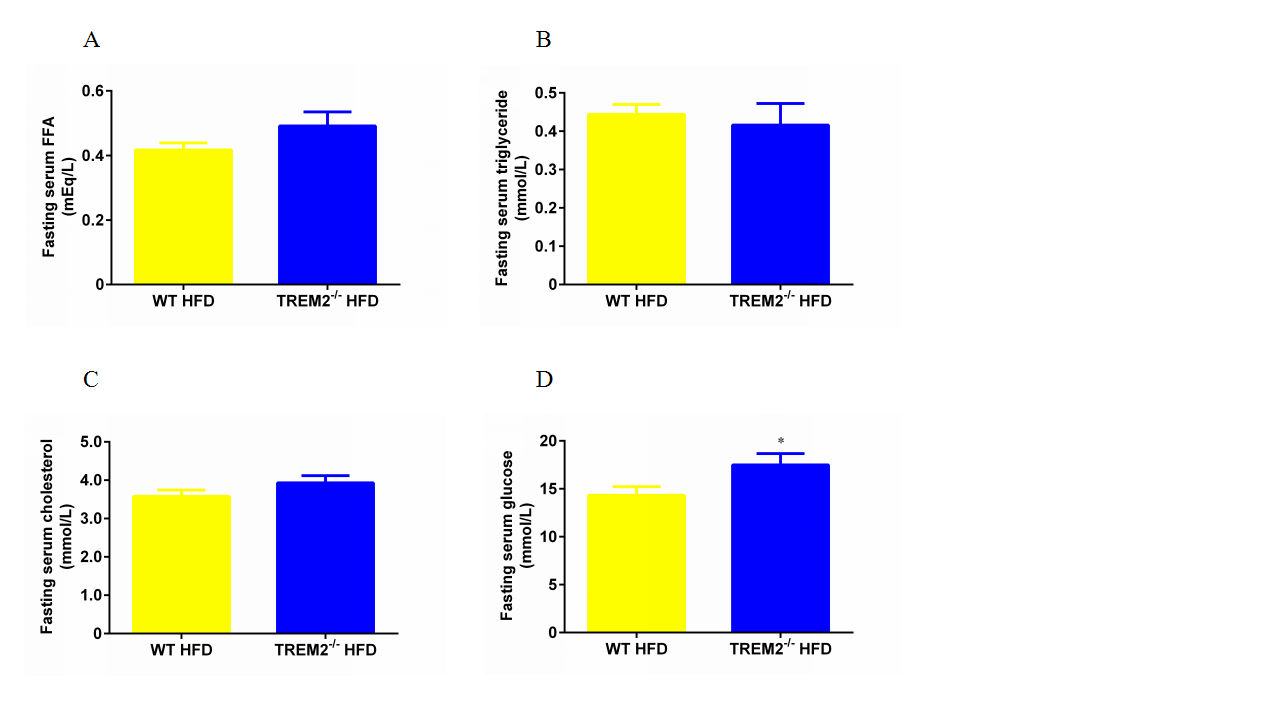

Supplement: Supplementary file 3 — Additional file 3: Figure S3. TREM2−/− mice demonstrated elevated fasting serum glucose under HFD. WT and TREM2−/− mice of C57BL/6 of 6 weeks (n = 13/group) were fed with HFD for 12 weeks. After HFD challenge, mice were sacrificed after 16 hours of fasting. Serum was collected and metabolic profiles were analyzed. Data are presented as mean ± SEM. *p < 0.05, **p < 0.01, ***p < 0.001, ****p < 0.0001. [file 12967_2019_2050_MOESM3_ESM.tif]

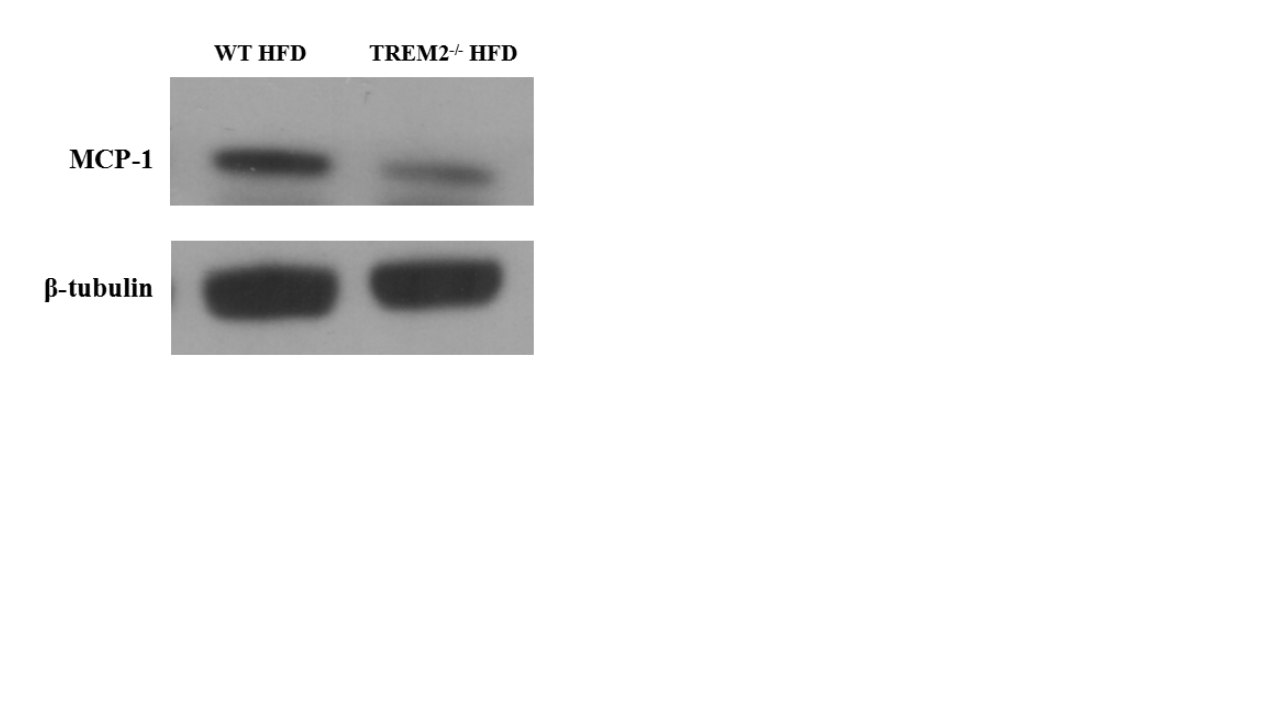

Supplement: Supplementary file 4 — Additional file 4: Figure S4. Adipocytes from TREM2−/− mice exhibited with down-regulated of MCP-1 level under HFD. WT and TREM2−/− mice of C57BL/6 of 6 weeks were fed with HFD for 12 weeks. After HFD challenge, mice were sacrificed and adipocytes were isolated for detecting MCP-1 protein level. [file 12967_2019_2050_MOESM4_ESM.tif]

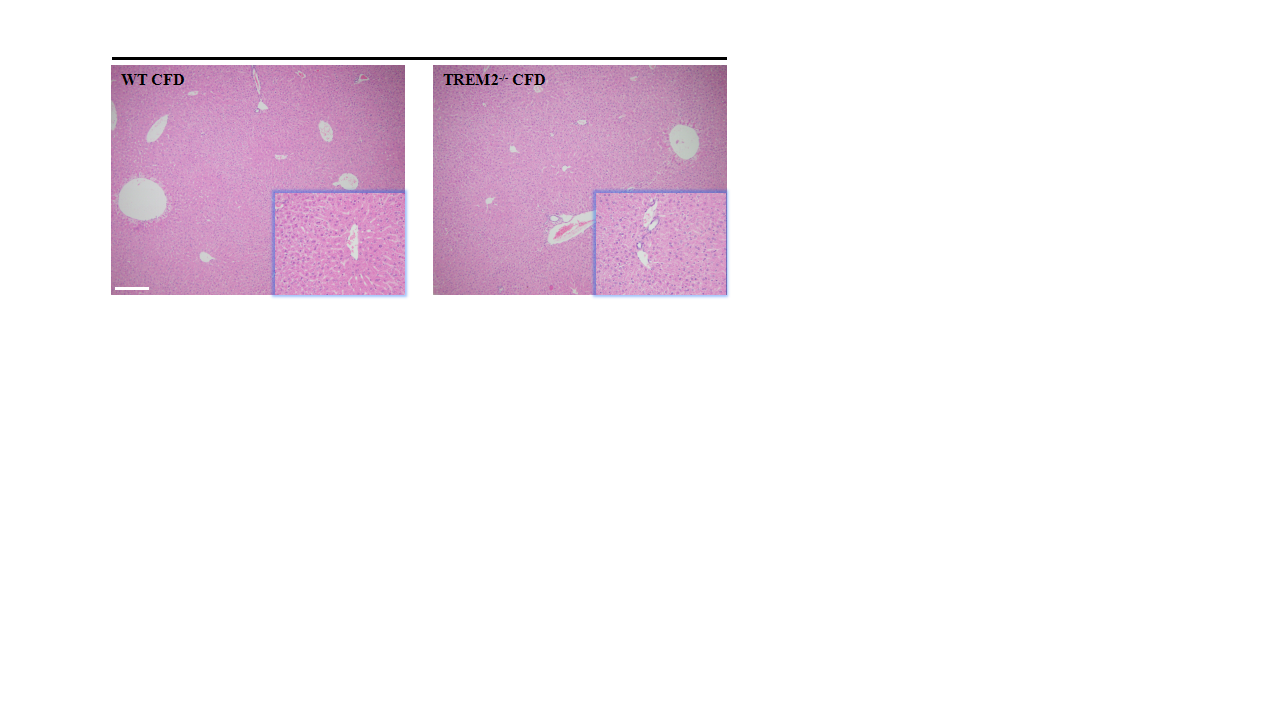

Supplement: Supplementary file 5 — Additional file 5: Figure S5. TREM2 deficiency fail to promote hepatic steatosis in mice of CFD. WT and TREM2−/− mice of C57BL/6 of 6 weeks were fed with CFD for 12 weeks. After CFD challenge, mice were sacrificed and hepatic steatosis was examined via H&E staining. Original magnification is 100× and 400× (within box at bottom right), scale bar = 200 μm. [file 12967_2019_2050_MOESM5_ESM.tif]
